# The Usefulness of Line-Field Confocal Optical Coherence Tomography in Monitoring Epidermal Changes in Atopic Dermatitis in Response to Treatment: A Pilot Study

**DOI:** 10.3390/diagnostics14161724

**Published:** 2024-08-08

**Authors:** Zuzanna Dryżałowska, Leszek Blicharz, Agnieszka Michalczyk, Jan Koscian, Małgorzata Maj, Joanna Czuwara, Lidia Rudnicka

**Affiliations:** Department of Dermatology, Medical University of Warsaw, 02-088 Warsaw, Poland; zdryzalowska@gmail.com (Z.D.); lblicharz@gmail.com (L.B.); adkaczorowska@gmail.com (A.M.); jan321koscian@gmail.com (J.K.); megi.maj@gmail.com (M.M.); lidiarudnicka@gmail.com (L.R.)

**Keywords:** atopic dermatitis, non-invasive imaging, line-field confocal optical coherence tomography, treatment monitoring, dupilumab

## Abstract

Background: Atopic dermatitis (AD) is the most common chronic inflammatory skin disease. Due to its high prevalence, considerable morbidity, and chronicity, there is a need for the accurate in vivo evaluation of treatment efficacy. Line-field confocal optical coherence tomography (LC-OCT) is a new emerging imaging technique able to perform a non-invasive, real-time examination of the epidermis and the upper dermis. LC-OCT may represent a promising tool in the diagnosis and treatment follow-up of chronic eczematous skin diseases with barrier defects. Objectives: We aimed to investigate the role of LC-OCT in the non-invasive monitoring of the treatment effect on five patients with severe atopic dermatitis during dupilumab treatment. Materials and Methods: LC-OCT imaging was performed on five patients (three women and two men) aged between 14 and 85 years old at the baseline and at 2, 4, and 6 weeks of treatment with dupilumab. The LC-OCT scans were performed at two sites, the lesional skin in the antecubital fossa and the extensor part of the arm, considered a control site on each patient for comparison. The captured images were later evaluated. Descriptive statistics and a *t*-test were used to compare the analyzed parameters over time and between involved atopic skin and clinically healthy skin. Results: The LC-OCT imaging was able to detect the difference in stratum corneum (SC) thickness and quality and epidermal thickness (ET) and the changes before and after treatment with high accuracy. The main findings include a significant reduction in the epidermal and stratum corneum thickness and decreased epidermal spongiosis and inflammation, with better quality of the stratum corneum indicating restoration of its tightness at both lesional and control sites. Conclusions: This study demonstrates that clinical improvement of affected and unaffected atopic skin under dupilumab treatment correlates with the LC-OCT findings. LC-OCT represents a novel, non-invasive tool examining the in vivo skin barrier and inflammation and can help to monitor the treatment efficacy among patients with atopic dermatitis in daily practice.

## 1. Introduction

Atopic dermatitis (AD) is a chronic and relapsing inflammatory dermatosis that usually begins in early childhood [1,2]. According to reports in the literature, AD affects more than 223 million people worldwide, of whom 43 million are children aged 1–4 [3]. The clinical picture of atopic dermatitis includes eczematous lesions in typical, age-characteristic locations, accompanied by intense itching [2]. Parents of the affected children often suffer from atopic diseases, such as atopic eczema, bronchial asthma, allergic rhinitis, or food allergies, indicating genetic inheritance [1,4].

The clinical presentation of atopic dermatitis changes with age [5]. In children under 2 years of age, the lesions typically have the morphology of acute eczema, with the presence of exudative papules located symmetrically on the face, the extensor surfaces of the limbs, and the trunk [6]. Children aged 2 to 12 years old usually present subacute eczema, with lesions predominantly present in the antecubital and popliteal fossae [6]. In adolescents over 12 years of age and adults, most severe lesions with the features of chronic or subacute chronic eczema are preferentially located on the face, neck, flexural areas, and upper back [6]. These lesions present a significant tendency towards lichenification and are prone to periodic exacerbations [6].

A diagnosis of atopic dermatitis is established based on the clinical manifestations. In clinical practice, the Hanifin and Rajka criteria are most frequently utilized, from which 3 out of 4 major criteria and 3 out of 23 minor criteria are required for AD diagnosis [7].

### 1.1. Pathogenesis of Atopic Dermatitis

The pathogenesis of atopic dermatitis is very complex and has not been fully explained yet. It is postulated that this disease develops in genetically predisposed people under the influence of a heterogeneous profile of environmental factors [8,9]. There are three main pathogenetic factors: epidermal barrier defects [10], abnormalities in the immune response [11], and microbial dysbiosis [12].

Epidermal barrier defects manifest, among other things, as disturbances in the structure of the skin’s lipid layer, impaired formation of tight junctions, and abnormal expression of proteins of the epidermal differentiation complex (EDC), including reduced production of filaggrin [8,13]. The effects of these disturbances lead to increased transepidermal water loss (TEWL), an increased surface pH, and increased penetration of allergens and irritating factors [14], which stimulate a Th2-dependent immune response and skin inflammation [9]. Depending on the phase of the disease analyzed and the subpopulation of patients, the Th17 and Th22 axes are also activated [11]. Defects of the epidermal barrier and skin inflammation are the basis of microbiome dysbiosis, manifesting as a reduction in its diversity and the selective expansion of *Staphylococcus aureus* [12]. This bacterium produces numerous virulence factors that intensify damage to the epidermal barrier and the expression of pro-inflammatory cytokines [12,15].

### 1.2. The Histology of Atopic Dermatitis

Histologically atopic dermatitis can be classified according to its chronicity. There are three main stages: acute, subacute, and chronic. Acute eczema is characterized by intercellular edema, with widening of the intercellular spaces in the epidermis and spongiotic microvesicle formation, which are filled with lymphocytes and Langerhans cells. The subacute phase presents a mild to moderate degree of spongiosis and lymphocytic exocytosis. This form is characterized by a variable degree of acanthosis and parakeratosis. Papillary dermal edema may be present. Chronic atopic dermatitis is represented by significant epidermal acanthosis, which may even show a psoriasiform pattern of hyperplasia with different degrees of parakeratosis on the surface. In this stage, fibrosis of the papillary dermis may be present, induced by constant rubbing and chronic inflammation [16].

### 1.3. Therapeutic Implications

The complexity of the pathogenesis of AD is responsible for the failure of many therapies, with some success of combined treatment, but severe cases are frequently either reluctant or recurrent after a short period of improvement. Classic treatment methods, including topical anti-inflammatory preparations, phototherapy, and immunosuppressive therapy, are associated with suboptimal selectivity and therefore show limited effectiveness and/or a relatively high risk of side effects [17]. Deepened knowledge regarding the immunopathogenesis of AD and progress in the field of biomedical engineering have enabled the development of new, selective biological drugs and small-molecule inhibitors of intracellular transmission pathways that have revolutionized the therapy of atopic dermatitis [17]. One example biological drug is dupilumab, a human monoclonal antibody, whilst the second group relies on JAK inhibitors [18].

### 1.4. Dupilumab

Dupilumab is an interleukin 4 (IL-4) receptor α-antagonist that acts by inhibiting IL-4 and IL-13 signal transduction through IL-4α subunit blockade [19]. IL-4 and IL-13 interleukins are responsible for the Th2 response; hence, dupilumab is considered to effectively reduce this immune reaction pathway [20]. It has been approved in the USA and Europe for treating moderate to severe AD [20]. It has been proven that dupilumab injections tend to improve clinical and patient-reported outcomes, such as the Eczema Area Severity Index (EASI) and the Dermatology Life Quality Index (DLQI) [19].

### 1.5. Clinical Scoring

The severity of atopic dermatitis is evaluated by using scores that incorporate both objective and subjective symptoms. Disease severity scales are used to evaluate whether the treatment goal has been achieved or the current treatment must be changed due to inefficiency. The most frequently used scores in atopic dermatitis are the Eczema Area and Severity Index (EASI), the SCORAD index, and Investigator’s Global Assessment (IGA) [21]. These scales have been tested and validated; they are commonly used in clinical practice even though they were generally designed for clinical trials [22]. Nevertheless, visual-based scoring systems tend to be subjective and hence inaccurate, as visual examination might be biased by clinician experience [21]. Considering these limitations, our objective was to develop a reliable evaluation method using image-based scoring using novel non-invasive imaging techniques. This approach aims to provide objective guidance for clinical decision-making, thereby preventing delayed and inconsistent therapeutic interventions.

### 1.6. Line-Field Confocal Optical Coherence Tomography

Line-field confocal optical coherence tomography (LC-OCT) is a novel non-invasive emerging technique that enables in vivo analysis of the skin [23]. It combines the principles of reflectance confocal microscopy (RCM) and optical coherence tomography (OCT) enabling skin visualization in both the vertical and horizontal planes and in three dimensions [24,25].

It provides real-time images at cellular resolution with a shorter penetration depth than OCT but a greater penetration depth in comparison to RCM [26]. LC-OCT has gained its value for the diagnosis of melanocytic and non-melanocytic skin tumors [26]. LC-OCT detection criteria have been created to differentiate between such skin lesions [23,27,28]. In recent years, non-invasive imaging techniques have also been used to diagnose inflammatory skin diseases, such as psoriasis, atopic dermatitis, or lichen planus [29]. LC-OCT skin imaging focuses on demonstrating the architectural composition of and changes in the epidermal layers, especially the stratum corneum (SC), the shape and visibility of the dermal–epidermal junction (DEJ), the vascular architecture, fluid accumulation, or inflammatory infiltrates [30,31,32].

## 2. Materials and Methods

### 2.1. Subjects and Biologic Treatment

This study was conducted according to the guidelines of the Declaration of Helsinki, and approval was obtained from the patients before examination. The inclusion criteria were a broad age (14–85 years), a diagnosis of AD based on the Hanifin–Rajka criteria, and the treatment regimen. Patients diagnosed with moderate to severe atopic dermatitis received treatment according to the treatment guidelines and underwent biologic treatment with the anti-IL-4Rα antibody dupilumab. Five patients were therefore included and analyzed with LC-OCT at the baseline and after 2, 4, and 6 weeks of treatment (*n* = 5).

### 2.2. Description of the Patients at Baseline

The patients selected for the study had atopic dermatitis that was moderate to severe at the baseline, as determined by a score of at least 16 on the Eczema Area and Severity Index (EASI; scores range from 0 to 72, with higher scores indicating greater severity) [33].

The patients’ age, sex, EASI and cDLQI/DLQI scores, and previous treatment are presented in Table 1. All the patients had atopic dermatitis since childhood. In all of them, the target lesions were in the antecubital fossa. The control area was the extensor surface of the upper arm. The dupilumab dosing for each patient as well as additional therapies are presented in Table 2.

### 2.3. Clinical Scoring

Visual-based scores such as the Eczema Area and Severity Index (EASI) were determined by a physician. In cases of atopic dermatitis, the following clinical signs were graded using a 4-point scale (0 = absent, 1 = mild, 2 = moderate, and 3 = severe): dryness, lichenification, excoriation, erythema, edema, and crusting [21,34]. Quality of life was assessed using the Children’s Dermatology Life Quality Index (cDLQI) and the Dermatology Life Quality Index (DLQI), which are used to measure the impact of skin diseases on the lives of patients according to their age [3].

### 2.4. The LC-OCT Image Acquisition Protocol

Since whole-body LC-OCT scanners do not exist, the selection of target lesions for small-area imaging was carefully made [21].

**Figure 1:**
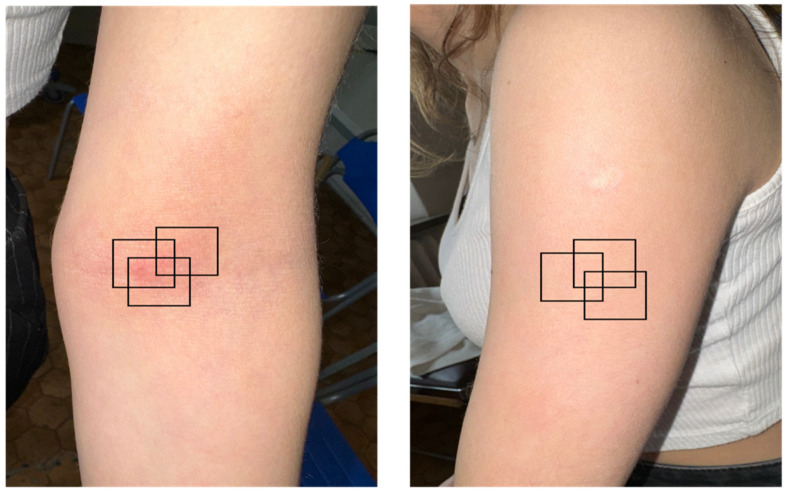
Selection of the target lesion and control site for line-field optical coherence tomography (LC-OCT). The scans were performed repetitively at the same lesional and non-lesional locations.

We utilized a clinically approved deepLive™ medical device, with a 3D stack size of 1.2 mm × 0.5 mm × 0.5 mm, a laser wavelength of 600–900 nm, and a penetration depth greater than 400 um. The lateral and axial resolution was <1.3 μm [35]. Images were acquired through vertical scans, with patients at rest prior to scanning. The measurement conditions, including location, patient position, and room temperature, were maintained. Repetitive imaging was conducted on the target lesion in the antecubital fossa and on clinically normal skin at the extensor site of the upper arm. Overview images of the body and specific skin areas ensured consistency in the measurement locations. The device was gently placed on the skin to avoid exerting pressure, and scales were not removed. The patients reported no discomfort during the examination. LC-OCT image data for five patients were collected. At the baseline and weeks 2, 4, and 6 of treatment, LC-OCT scans were performed at the two abovementioned sites, with 3 photos per each site on each patient (Figure 1).

### 2.5. Dermoscopy Examination

LC-OCT examination in in vivo mode enables the simultaneous acquisition of dermoscopic images. The dermoscopic examination field covers a circular area with a diameter of 2.5 mm and a resolution of 5 µm.

### 2.6. The Imaging Parameters

A total of 240 selected lesional LC-OCT images were evaluated. In each patient, 3 photos per target lesion in the antecubital fossa and 3 photos per the clinically normal skin at the extensor site of the upper arm were taken and analyzed at weeks 0, 2, 4, and 6.

The imaging parameters that were taken into consideration included stratum corneum thickness and quality and epidermal thickness and changes since atopic dermatitis barrier dysfunction is the hallmark of atopic skin.

The measures were performed by the same scientist (ZD) using an on-screen ruler always at standard magnification. The greatest width of the stratum corneum and the epidermal thickness was measured, and the average value was calculated from 3 acquisitions at the same site. The stratum corneum was measured from the surface to the stratum granulosum, whereas the epidermal thickness was measured from the surface to the bottom of rete ridges.

### 2.7. Statistics

Numerical variables are expressed as means ± standard deviation. An evaluation was made after checking whether the parameters were normally distributed. The stratum corneum thickness and the epidermal thickness at different times (week 0, week 2, week 4, and week 6) were calculated and compared between the two groups (lesional and clinically healthy skin) using a *t*-test. A value of *p* < 0.05 was accepted for the significance level of the tests.

## 3. Results

### 3.1. Atopic Dermatitis in LC-OCT

**Figure 2:**
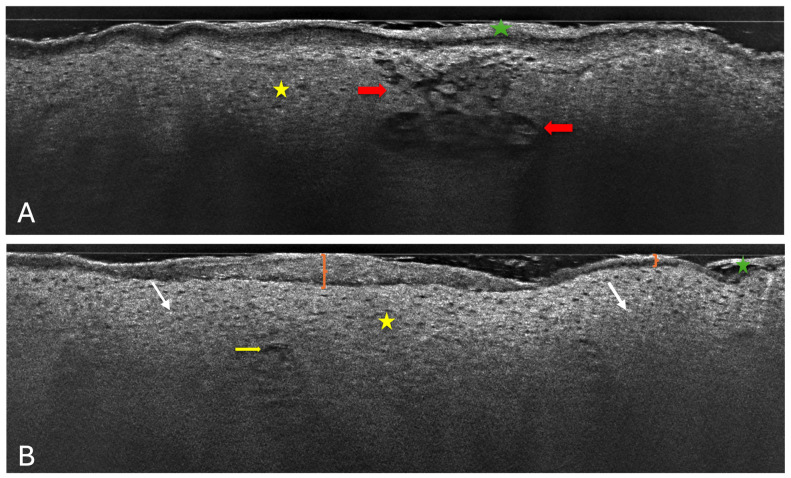
Atopic dermatitis. (**A**) Vertical LC-OCT section shows an unevenly thickened and desquamative stratum corneum (green star) with a focal grainy appearance due to parakeratosis over the vital epidermis, with a disrupted honeycomb pattern (yellow star), and dark roundish areas containing grouped, scattered keratinocytes (red arrows) separated by spongiosis, leading to spongiotic intraepidermal vesicle formation. (**B**) Vertical LC-OCT section shows a thick and irregular cornified layer (orange brackets) covered with serum crust (green star), a thick spinous layer corresponding to epidermal hyperplasia, with spaces between the keratinocytes due to spongiosis (yellow star), intraepidermal microvesicles (yellow arrow), and inflammatory cells entering the vital epidermis as an indication of exocytosis (white arrows).

LC-OCT allows for recognition and measurement of the structures located in the epidermis and the dermis. In the vertical view (Figure 2), the top structure is the stratum corneum, which appears as a hyper-reflective band [24]. The key characteristic of atopic dermatitis is a thickened, irregular, and disrupted stratum corneum (SC) [26]. This is indicative of the impaired skin barrier function typical of AD. Located underneath stratum granulosum and stratum spinosum are composed of roundish keratinocytes with hyporeflective nuclei. In AD, the regular honeycomb pattern is altered [30]. This may be due to spongiosis, which refers to intercellular edema leading to a spongy appearance [34]. It can be visualized as dark spaces between cells and is a hallmark of acute eczema [36]. Additionally, the skin of AD patients shows greater inflammatory infiltrates, which are visible as bright spots in the dermis [36]. Besides irregular SC, alteration of the regular honeycomb pattern, and spongiosis, another characteristic of AD is epidermal thickening [26]. Just below, the dermal–epidermal junction (DEJ) should appear as a hyporeflective line, which marks the border between the epidermis and the dermis. In healthy skin, the junction has a wavy shape due to the presence of dermal papillae. However, in cases of AD, the DEJ can be very hard to distinguish, as inflammatory cells infiltrate both the dermis and the epidermis [29]. At the bottom part of an LC-OCT image, the dermis is visible. It is displayed as a hyperreflective signal due to its many collagen and elastic fibers, which can be described as bright and wavy [24]. The vessels are hyporeflective, long, linear structures. The most typical vessel alterations in AD include increased size and dilation, reflecting the inflammatory nature of the disease [37].

### 3.2. Stratum Corneum Thickness

**Figure 3:**
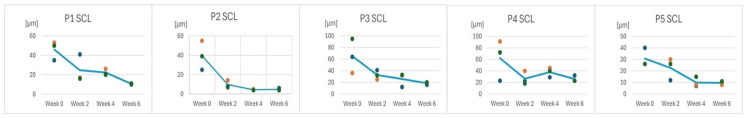
Changes in the stratum corneum at lesional site SC_L_ during dupilumab treatment in five patients with atopic dermatitis. Each dot represents one measurement, blue line represents the mean of those values.

During the six-week treatment period with dupilumab, the patients exhibited a significant reduction in stratum corneum thickness at the lesional sites, which can be seen in Figure 3. At baseline, we observed a greater thickness of the stratum corneum in the target lesions SC_L_ among all patients compared to the measurements taken after six weeks of treatment. The initial measurements at week 0 revealed a mean thickness of 48.67 μm (±22.42 μm). By week 2, its thickness had significantly decreased to a mean of 23.27 μm (±11.17 μm), with a *p*-value < 0.001. Continued treatment further reduced its thickness, with the week 4 measurements showing a mean of 20.07 μm (±13.15 μm) (*p*-value < 0.001). At week 6, the mean thickness was substantially lower, at 13.93 μm (±7.84 μm), with a *p*-value < 0.001. These findings indicate that dupilumab effectively reduces the stratum corneum thickness at lesional sites, with notable improvements observed as early as the second week and continuing through the treatment period.

**Figure 4:**
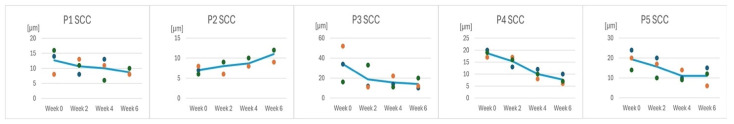
Changes in stratum corneum at control site SC_C_ during dupilumab treatment in five patients with atopic dermatitis. Each dot represents one measurement, blue line represents the mean of those values.

**Figure 5:**
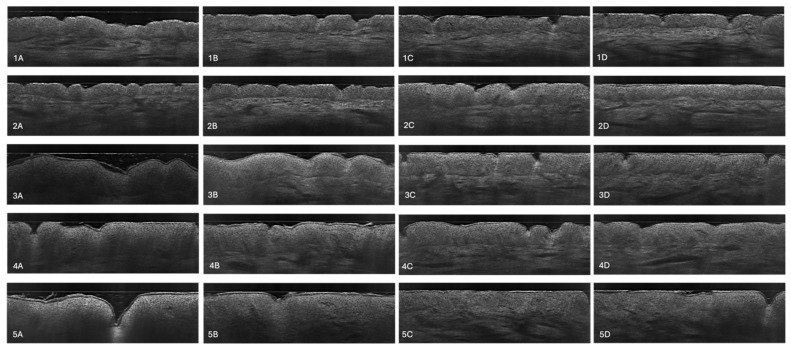
LC-OCT-guided monitoring of biologic therapy in atopic dermatitis. The vertical scans of the control skin on the upper arm were collected in the course of therapy. Each row represents each patient. The images in the first column (**1A**–**5A**) were taken before treatment, the second column (**1B**–**5B**) represents skin after first 2 weeks after the treatment implementation, the third column (**1C**–**5C**) shows skin after 4 weeks of dupilumab, and the last column represents skin after 6 weeks of treatment (**1D**–**5D**).

Comparatively, the stratum corneum at the control site SC_C_ also displayed an increased baseline thickness. During the six-week treatment period with dupilumab, the patients experienced a reduction in stratum corneum thickness at the control site, although the changes were less pronounced compared to those at the lesional sites (Figure 4). The reduction in the stratum corneum at the control site can be seen in Figure 5, where LC-OCT images of the skin on the upper arm during dupilumab treatment are shown.

**Figure 6:**
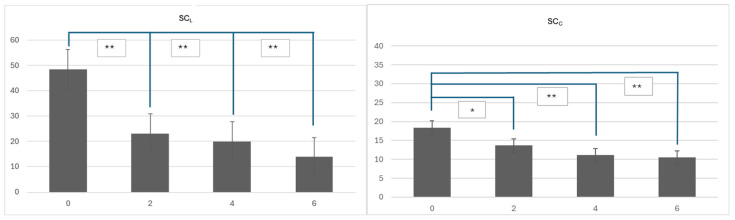
Changes in the stratum corneum at the lesional site (SC_L_) and the stratum corneum at the control site (SC_C_) during treatment. * *p* = 0.01, ** *p* = 0.001.

Starting from an initial mean thickness of 18.33 μm (±11.43 μm), there was a gradual but consistent decrease across the weeks. By week 2, its thickness had decreased to 13.67 μm (±6.36 μm), though this change was not statistically significant (*p*-value = 0.183). More significant reductions were observed by week 4, with its thickness further reduced to 11.07 μm (±3.68 μm) (*p*-value = 0.008), and by week 6, it had reduced to 10.47 μm (±3.66 μm) (*p*-value = 0.024). The calculated means and the population standard deviations provide further insight into the distribution and variability of the data at each time point. The largest average value was observed at week 0, with a gradual decrease in the following weeks. This trend was accompanied by a decrease in variability, as shown by the decreasing standard deviations over time. These findings demonstrate that dupilumab effectively reduced the stratum corneum thickness at the control sites, particularly after the fourth week of treatment. The reductions in SC_C_ and SC_L_ thickness during dupilumab treatment and their significance can be seen in Figure 6.

**Figure 7:**
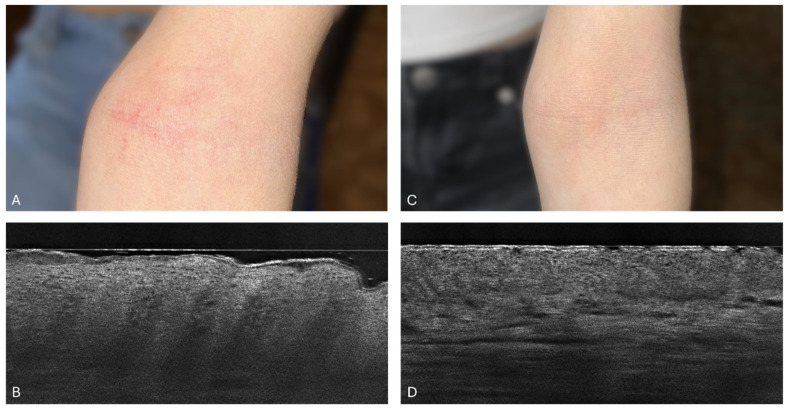
Patient 1: Clinical photograph of the patient (**A**) and line-field optical coherence tomography (LC-OCT) image (**B**) before treatment implementation. LC-OCT image (**B**) of the antecubital fossa revealed a thick and disrupted stratum corneum, as well as thickening of the epidermis and extended dermal papillae. Clinical photo of the patient (**C**) and LC-OCT image (**D**) after 6 weeks of dupilumab treatment. LC-OCT image (**D**) from the antecubital fossa revealed a significant reduction in the stratum corneum and epidermis thickness.

In an examination of a 14-year-old girl with acute atopic dermatitis, SC_L_ thickness measurements were tracked over six weeks following dupilumab treatment. Initially, by week 2, the mean SC_L_ thickness was 24.7 μm (±11.6) from the baseline of 46 μm (±7.9 μm), showing a small reduction. However, from week 4 onwards, more noticeable reductions were observed. By week 4, its thickness had decreased to 22.3 μm (±2.6 μm), and by week 6, it had further reduced to 10.7 μm (±0.5 μm). These changes demonstrate the effectiveness of dupilumab in reducing SC_L_ thickness, with improvements particularly evident from the fourth week of treatment. Figure 7 in this study illustrates clinical and LC-OCT images of the skin before and after 6 weeks of dupilumab treatment, clearly showing the treatment’s effectiveness.

**Figure 8:**
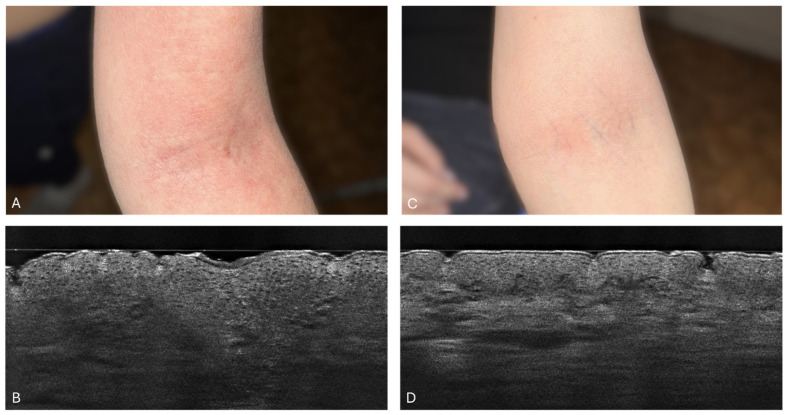
Patient 2: Clinical photograph of the patient (**A**) and line-field optical coherence tomography (LC-OCT) image (**B**) before treatment implementation. LC-OCT (**B**) image of the antecubital fossa revealed an irregular, disrupted, and thick stratum corneum, as well as thickening of the epidermis. Clinical photograph of the patient (**C**) and LC-OCT image (**D**) after 6 weeks of dupilumab treatment. LC-OCT (**D**) image of the antecubital fossa revealed a significant reduction in the stratum corneum and epidermal thickness. The SC appears more regular, flat, and smooth.

In the analysis of the SC_L_ thickness in a 34-year-old woman with acute atopic dermatitis, changes were observed over the treatment period. Initially, at week 0, the SC_L_ thickness was measured at 39.7 μm (±12.3 μm). By week 2, the SC_L_ thickness reduced to 9.7 μm (±3.1 μm). Subsequent measurements showed that the thickness stabilized, at 4.3 μm (±0.5 μm) by week 4 and at 4.7 μm (±0.9 μm) by week 6. These results suggest that the most substantial reduction occurred quickly by week 2, with the thickness remaining relatively stable thereafter. Figure 8 illustrates the clinical and LC-OCT examination of this patient, highlighting the effectiveness of the treatment over time.

**Figure 9:**
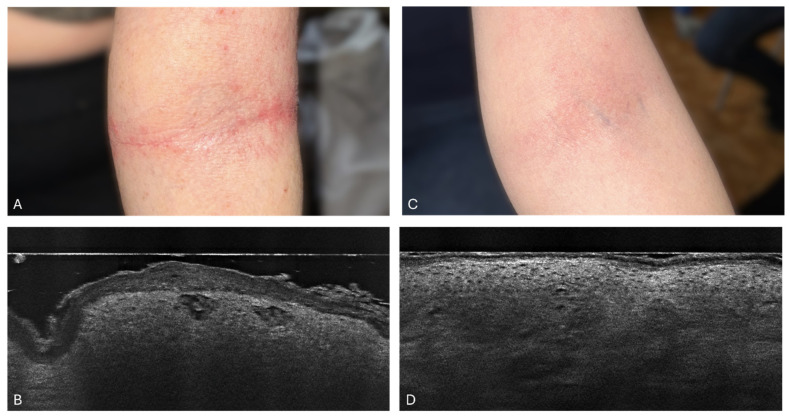
Patient 3: Clinical photograph of the patient (**A**) and LC-OCT image (**B**) before treatment. LC-OCT (**B**) image of the antecubital fossa revealed an irregularly thickened SC, lichenification, spongiotic vesicles, and inflammatory changes in the epidermis. Clinical photograph of the patient (**C**) and LC-OCT image (**D**) after 6 weeks of dupilumab treatment. LC-OCT (**D**) image of the antecubital fossa revealed a significant reduction in SC thickness, as well as epidermis thickness. The intensity of spongiosis is also reduced. The dermal–epidermal junction (DEJ) is visible, showing a border between the epidermis and dermis.

In a study involving a 32-year-old woman with exacerbated atopic dermatitis, SC_L_ thickness measurements were tracked over a six-week period, as detailed in Figure 3. Initially, at week 0, the SC_L_ thickness was 65 μm (±24.1 μm). By week 2, it reduced to 32.7 μm (±6.5 μm). The trend of a reduction continued with the SC_L_ thickness further decreasing to 25.7 μm (±9.7 μm) at week 4 and reaching 18.7 μm (±1.9 μm) by week 6. This pattern suggests a consistent reduction in the SC_L_ thickness across the weeks, with the greatest apparent reduction occurring between week 0 and week 6. The smallest standard deviation was noted at week 6, indicating more consistent scores among the subjects by that time. Figure 9 compares the baseline and week 6 condition of the skin, showcasing clinical photography and LC-OCT images to illustrate these changes.

**Figure 10:**
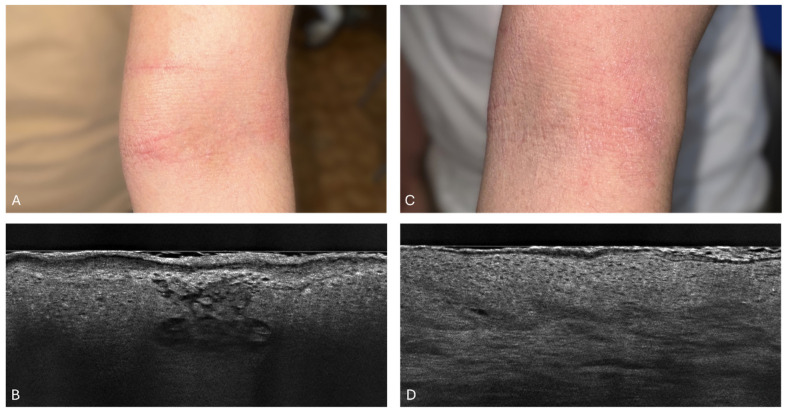
Patient 4: Clinical photograph of the patient (**A**) and LC-OCT image (**B**) before treatment. LC-OCT (**B**) image of the antecubital fossa revealed an irregularly thickened SC, intense spongiosis, vesicles, and inflammation in the center of the epidermis. Clinical photograph of the patient (**C**) and LC-OCT image (**D**) after 6 weeks of dupilumab treatment. LC-OCT (**D**) image of the antecubital fossa revealed a significant reduction in SC thickness, as well as epidermal thickness. The disappearance of spongiotic and inflammatory vesicles can be seen with the restoration of the honeycomb appearance of the epidermis.

In the study of a 24-year-old man with atopic dermatitis, SC_L_ thickness was monitored over 6 weeks. Initially, at baseline, the SC_L_ thickness was recorded at 62 μm (±28.6 μm). By week 2, this thickness reduced to 26.7 μm (±9.6 μm), and by week 6, it further decreased slightly to 26 μm (±4.3 μm), indicating a general downward trend. Interestingly, week 4 showed a temporary increase in the SC_L_ thickness to 38 μm (±6.7 μm), suggesting a transient exacerbation of the skin’s condition. Despite these fluctuations, the overall pattern highlights a reduction in the SC_L_ thickness from the baseline to the end of the study period, with some variability in the interim weeks. Figure 10 illustrates these changes through clinical and LC-OCT images of the patient’s skin.

**Figure 11:**
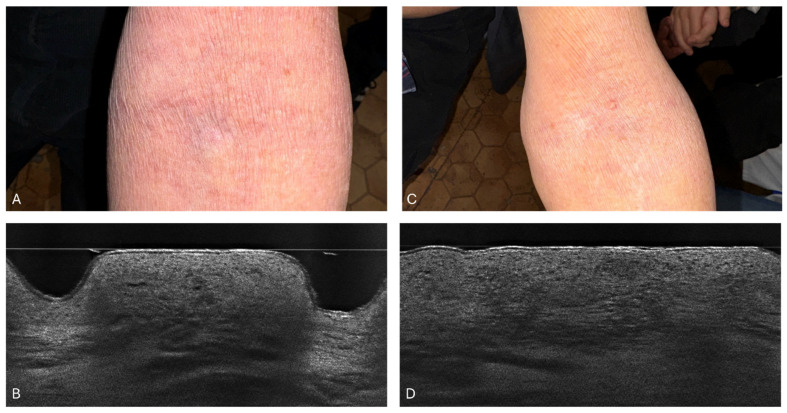
Patient 5: Clinical photograph of the patient (**A**) and LC-OCT image (**B**) before treatment. LC-OCT (**B**) image of the antecubital fossa revealed lichenification and scattered spongiotic and inflammatory vesicles. Clinical photograph of the patient (**C**) and LC-OCT image (**D**) after 6 weeks of dupilumab treatment. LC-OCT (**D**) image of the antecubital fossa revealed a significant reduction in SC and epidermal thickness and irregularity and decreased lichenification and inflammation, with restoration of the honeycomb appearance of the epidermis.

In a study performed on an 85-year-old man with atopic dermatitis, the SC_L_ thickness was closely monitored over several weeks. Initially, at week 0, the SC_L_ thickness was 30.7 μm (±6.6 μm). By week 2, it slightly decreased to 22.7 μm (±7.7 μm). This downward trend continued to week 4 and week 6, with the mean SC_L_ thickness values reaching 10 μm (±3.6 μm) and 9.7 μm (±1.2 μm), respectively. This progression suggests a gradual thinning of the SC_L_ over the course of the study, marking a positive response in later weeks. Figure 11 illustrates these changes through clinical and LC-OCT images, documenting the skin’s condition before and after the treatment period.

### 3.3. Stratum Corneum Disturbances

**Figure 12:**
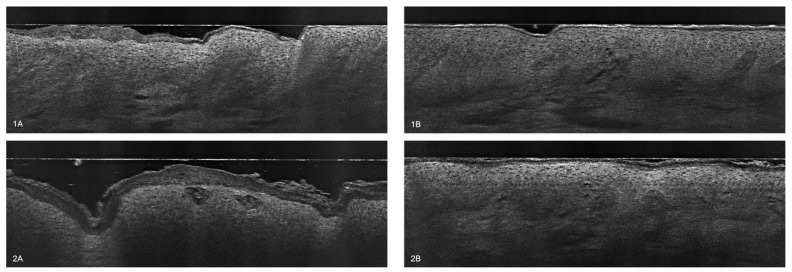
The stratum corneum quality changes before (**1A**,**2A**) and after 6 weeks of treatment (**1B**,**2B**).

Figure 12 shows the changes in the stratum corneum from the baseline to week 6. The SC became thinner, regular, flat, and smoother after the treatment (1B, 2B), indicating normalization of its function and restoration of its barrier properties [38]. The tightness of the SC appears to be greater in the right-hand panel of the LC-OCT examination (1B, 2B), which may reduce transepidermal water loss and the penetration of pathogens and allergens [14]. Another difference is the extent of SC exfoliation, which is much greater at week 0 (1A, 2A). The level of graininess also differs—it is more prominent in the lesional skin before treatment (1A, 2A), indicating a greater extent of parakeratosis [16]. Lichenification, which arises due to chronic scratching and leads to thickened and leathery skin, makes the skin markings more pronounced [16]. This characteristic is more visible in the left-hand images (1A, 2A).

The dermal–epidermal junction is more visible after 6 weeks of treatment, especially in LC-OCT image 2B. This indicates that the border between the epidermis and the dermis is more defined, as inflammatory infiltrates are reduced [16]. When the level of inflammation is high, the typical honeycomb pattern of the stratum spinosum is altered, making it hard to separate from the dermis [29]. The DEJ of the treated skin tends to be flatter in comparison with lesional skin on the left (2A). Acanthosis, which is a form of irregular epidermal thickening due to reactive changes in response to inflammation and increased sizes and numbers of keratinocytes, makes the epidermis thicker and the rete ridges wider, influencing the regular wavy shape of the DEJ [16]. Another difference worth mentioning is the presence of spongiosis, a hallmark of acute and subacute atopic dermatitis that refers to intercellular edema in the epidermis [16]. A spongy appearance is more visible before the treatment (1A, 2A).

### 3.4. The Epidermal Thickness

**Figure 13:**
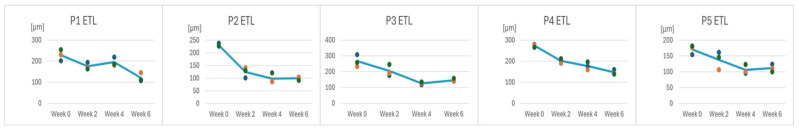
Changes in epidermal thickness at the lesional site ET_L_ during dupilumab treatment in five patients with atopic dermatitis. Each dot represents one measurement, blue line represents the mean of those values.

This study also assessed the reduction in epidermal thickness at the lesional site ET_L_ in the patients over a six-week period of dupilumab treatment, with measurements taken at weeks 0, 2, 4, and 6. The data are presented in Figure 13. Initially, at week 0, its thickness ranged from 154 to 307 μm, with a mean value of 233.93 μm (±40.82 μm). By week 2, its mean thickness had decreased to 168.4 μm (±38.12 μm), and by week 4, it further reduced to 140.27 μm (±41.07 μm). At the end of the treatment period, by week 6, the mean epidermal thickness was diminished to 124.87 μm (±21.56 μm).

The statistical analysis using paired Student’s *t*-tests demonstrated significant reductions between week 0 and each subsequent week: week 2 (*p*-value < 0.001), week 4 (*p*-value < 0.001), and week 6 (*p*-value < 0.001). Overall, the data indicate a marked reduction in epidermal thickness at lesional site ET_L_ over the six-week treatment period, with the first significant difference occurring after the second week. This pattern suggests that dupilumab is highly effective in reducing epidermal acanthosis or hyperplasia, with sustained improvements observed throughout the treatment duration.

**Figure 14:**
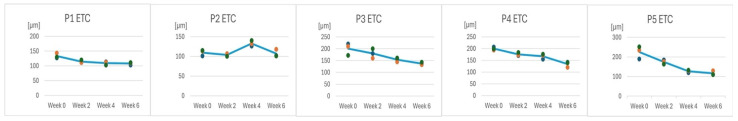
Changes in the epidermal thickness at the control site ET_C_ during dupilumab treatment in five patients with atopic dermatitis. Each dot represents one measurement, blue line represents the mean of those values.

During the six-week treatment period with dupilumab, the patients exhibited a significant reduction in the epidermal thickness of the control site ET_C_. The reduction pattern can be seen in Figure 14. The initial measurements at week 0 indicated a mean thickness of 173.80 μm (±46.98 μm). By week 2, the mean thickness had decreased to 149.60 μm (±34.56 μm), showing a statistically significant reduction (*p*-value = 0.005). Further reductions were observed at week 4, with a mean thickness of 138.13 μm (±21.33 μm), which was also statistically significant (*p* = 0.007). By the end of the tracked treatment period at week 6, the mean thickness had reduced to 120.53 μm (±14.80 μm), representing a substantial decrease (*p*-value < 0.001). These results demonstrate that dupilumab effectively reduced the epidermal thickness of the control site ET_C_ as well, with notable improvements observed as early as the second week and continuing throughout the treatment duration.

**Figure 15:**
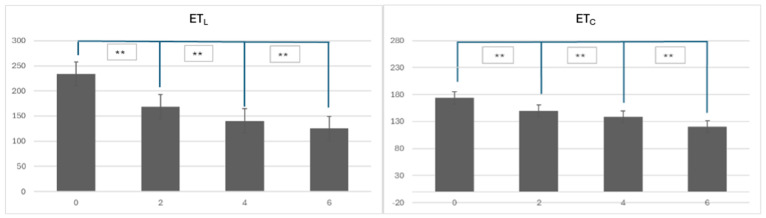
Changes in epidermal thickness at the lesional site (ET_L_) and epidermal thickness at the control site (ET_C_) during treatment. ** *p* = 0.001.

The reduction in ET_C_ and ET_L_ thickness during dupilumab treatment and their significance can be seen in Figure 15.

During the course of dupilumab treatment for Patient 1, epidermal thickness measurements were recorded. At the start of the treatment (week 0), the mean epidermal thickness was 228.67 μm (±21.25 μm). By week 2, the mean decreased to 175.67 μm (±13.27 μm). At week 4, the mean was 194.67 μm (±17.33 μm). By week 6, a reduction to 121.00 μm (±17.05 μm) was observed. In summary, while the epidermal thickness fluctuated in the early weeks of treatment, a notable reduction was observed by week 6, highlighting the effectiveness of the dupilumab treatment over the six-week period.

For Patient 2, epidermal thickness was measured during treatment, demonstrating changes over time. At week 0, its mean thickness was 230.33 μm (±5.44 μm). By week 2, the mean had decreased to 124.00 μm (±16.87 μm). At week 4, the mean further decreased to 98.00 μm (±16.27 μm). Finally, at week 6, the mean thickness was 99.33 μm (±5.91 μm). These findings highlight a reduction in the epidermal thickness as treatment progressed, indicating the treatment’s effectiveness for Patient 2.

The initial mean thickness at week 0 for Patient 3 was 266.33 μm (±30.65 μm). By week 2, the mean decreased to 204.00 μm (±30.24 μm). At week 4, the mean thickness dropped further to 126.00 μm (±6.53 μm). By week 6, the mean was 145.67 μm (±8.01 μm). These findings highlight a reduction in the epidermal thickness by weeks 4 and 6, indicating the effectiveness of the treatment over time. This pattern of a reduction in the epidermal thickness by weeks 4 and 6 was also observed in Patient 1, demonstrating the consistent effectiveness of the treatment in these patients.

At baseline, Patient 4 exhibited the thickest epidermis compared to the other patients, with a mean value of 274.00 μm (±5.72 μm). By week 2, its thickness had decreased to a mean of 200.67 μm (±8.58 μm). At week 4, the mean thickness was further reduced to 176.67 μm (±15.15 μm). By week 6, its thickness reached a mean of 146.67 μm (±9.46 μm). This consistent pattern of a reduction in epidermal thickness over time indicates the treatment’s effectiveness for Patient 4, similar to the trends observed in Patient 2.

For Patient 5, the treatment’s impact on epidermal thickness was monitored. Initially, at week 0, its mean thickness was the lowest compared to that of the other patients, probably due to his age of 85, with a mean value of 170.33 μm (±11.73 μm). By week 2, its thickness decreased to 137.67 μm (±23.21 μm). A subsequent reduction was observed by week 4, where the mean thickness dropped to 106.00 μm (±12.19 μm). At week 6, its thickness was 111.67 μm (±9.81 μm), indicating a continued trend of reduction.

## 4. Discussion

To the best of our knowledge, this is the first study that describes observing the moderate to severe skin changes in AD patients undergoing dupilumab treatment with LC-OCT. It was recently described that AD is characterized by a disrupted and thickened stratum corneum layer, increased epidermal thickness, enhanced lymphocyte infiltrates with the predominance of Th2 cells, vasodilation, and dermal edema [39]. This study demonstrates that within 6 weeks of dupilumab treatment, an overall improvement was observed in these characteristics in lesional skin with LC-OCT imaging compared to those at the baseline. The thickness of both the stratum corneum and the epidermis decreased significantly after the first 2 weeks of treatment. The quality of the stratum corneum, the epidermis, and the dermis also changed during the 6-week treatment follow-up. The stratum corneum became better organized, smoother, and tight. The extent of exfoliation and graininess became smaller, indicating reduced parakeratosis and desquamation. Lichenification became less pronounced, whereas the dermal–epidermal junction became more visible and flatter. Moreover, the intensity of spongiosis and intercellular edema was reduced. This study also reports a significant improvement in the skin and its normalization within the 6-week treatment monitoring, as in the stratum corneum, a significant reduction was described after 4 weeks of treatment, whereas the reduction in epidermal thickness was significantly faster, occurring after the first 2 weeks. Dupilumab’s effects extend beyond affected areas to include uninvolved atopic skin, demonstrating that this treatment helps to normalize Th2 response imbalance [40]. This finding underscores that AD is a systemic disease impacting the entire skin.

LC-OCT devices are convenient to use and provide easy access to the majority of sites on the body. They combine the capabilities of RCM in the context of its cellular resolution (~1 µm) and the advantages of OCT in terms of its penetration depth. Real-time display of vertical and horizontal sections, as well as 3D images, is combined with dermoscopy-like surface images acquired in parallel [26]. LC-OCT reveals the internal morphology of the skin at the cellular level by quantifying the intensity of light backscattered by tissue microstructures. Although variations in the tissue refractive index are less specific than histology, LC-OCT offers significant advantages, as it provides three-dimensional visualization in real time, eliminating the need for biopsy.

The usage of new emerging visualization techniques will enable a better understanding of the atopic dermatitis-disrupted skin barrier and its restoration during treatment [29]. LC-OCT can be used to monitor early inflammation and skin impairment prior to visible flare-ups to optimize treatment. In more complex situations, non-invasive imaging techniques can be used to identify the most accurate biopsy site for diagnostic purposes.

Advancements in the image analysis software significantly enhance the quantification of skin metrics such as the layer thickness, keratinocyte atypia, and DEJ undulation, thereby improving the diagnostic accuracy within and treatment monitoring of inflammatory skin diseases [41]. In our study, we have shown that LC-OCT is a technology that can image characteristics that were previously identified by ex vivo histology.

LC-OCT allowed Verzi et al. to recognize the main microscopic features of plaque psoriasis, atopic eczema, and lichen planus. Their non-invasive examination showed a thickened and disrupted stratum corneum, as well as a disorganized honeycomb pattern, spongiosis, and dark, roundish areas containing scattered keratinocytes in the epidermis. They described LC-OCT as a promising tool in biopsy guidance, follow-up, and treatment monitoring [29].

Manfredini et al. performed a study of dupilumab treatment monitoring in atopic dermatitis with the usage of dynamic optical coherence tomography (D-OCT). Their results align with the conclusion that we reached using LC-OCT, as they noticed a decrease in epidermal thickness and an improvement of epidermal barrier defects in the lesional skin. Their study described an improvement of clinically healthy skin in terms of collagen remodeling and inflammation [42]. Nevertheless, the aspects of reducing the stratum corneum thickness and improving its quality are not mentioned.

Ha-Wissel et al. presented a case study of OCT monitoring of biologic therapy in both psoriasis and atopic dermatitis. The eczema lesions exhibited a higher epidermal thickness, increased dermal vascular density, and a higher vessel count compared to uninvolved skin. They described a response to biologic therapy according to a reduced epidermal thickness and normalization of the vascular network. However, no significant changes were detected at the control sites [36].

The usage of LC-OCT for non-invasive examination of the epidermis and the upper dermis during the treatment of psoriasis was performed by Orsini et al. They performed LC-OCT imaging of moderate–severe plaque psoriasis before and after 8 weeks of treatment. In their study, the images were segmented by artificial intelligence, and the thickness of the SC and the epidermis, as well as the undulation of the dermal–epidermal junction, was evaluated [41]. Their study is a perfect example of using digital analysis to help in the recognition and quantification of microscopic criteria, which showed a reduction in all these parameters during the treatment follow-up.

In 2023, Donelli et al. summarized all the available data on the use of LC-OCT for inflammatory and infectious diseases. Based on 14 papers, they concluded that LC-OCT can reveal architectural changes in the skin, and these architectural criteria are mainly what make LC-OCT valid and useful. They seem to have proven that LC-OCT can be used as guidance for clinical diagnosis and thus reduce histological investigations. LC-OCT can highlight different epidermal layers, their alternations, and changes in their thickness (e.g., hyperkeratosis, acanthosis), the shape of the DEJ (papillomatosis), and the vascular architecture (vasodilation and shape of the vessels), as well as the presence of hyporeflective areas in the dermis that correlate with dense inflammatory infiltrates. They believe that image analysis software and artificial intelligence may aid in precisely identifying the subtypes of inflammatory cells (i.e., eosinophils, neutrophiles, and lymphocytes). Therefore, the development of advanced postprocessing software will help identify and characterize inflammatory infiltrates [30].

In vivo LC-OCT evaluation is a promising diagnostic method not only in AD. This non-invasive form of examination has been also proven useful in different epidermal disorders. Stefani et al. described its usefulness in supporting the diagnosis of Hailey–Hailey disease, as it allows us to recognize the main microscopic clues behind this disease [43].

Moreover, it has been described by Cappilli et al. that in vivo evaluation with LC-OCT provides practical clues in the identification of cutaneous vascular lesions, especially when the dermoscopic and clinical features are unclear. They suggest a strong correlation between the LC-OCT morphology and histopathological criteria for cutaneous vascular lesions [44].

A significant constraint of our study lies in the absence of histological images. However, the measurements we conducted and the qualitative changes we observed in the atopic skin at identical locations and on the same patients confer significant reliability to our study. It is also important to note that histology only examines a small piece of tissue, whereas LC-OCT images encompass a larger section of the skin in real time. Consequently, we do not consider the lack of a histopathological correlation to be a limitation concerning the results obtained from LC-OCT. The objective of this research was to demonstrate the efficacy of LC-OCT as a non-invasive tool monitoring the superficial skin changes.

## 5. Conclusions

Line-field confocal optical coherence tomography is a non-invasive skin imaging technique that becomes a useful tool for the diagnosis and treatment monitoring of many skin diseases, including inflammatory conditions. Its objective nature provides significant benefits, enhancing our understanding of the pathophysiology of atopic dermatitis (AD) and the role of the skin barrier. In this study, we evaluated the usage of LC-OCT in treatment monitoring for atopic dermatitis. We showed that dupilumab significantly reduces the stratum corneum and epidermal thickness at 6-week treatment follow-up in lesional and uninvolved skin. Moreover, it positively influences the quality of the skin barrier in the lesional skin. We believe that LC-OCT examination offers a valuable tool for assessing the skin barrier’s response to immunotherapy. Additionally, LC-OCT may soon be used to monitor early inflammation and skin damage before clinically visible flare-ups, thereby being helpful for optimizing the treatment strategies.

## Figures and Tables

**Table 1:** Patient characteristics before dupilumab treatment.

	Age	Sex	EASI	cDLQI/DLQI	Previous Treatment
Patient 1	14	F	20.4	12	oral cyclosporine, short episodes of oral glicocorticosteroids, oral antihistamines, topical steroids, and emollients
Patient 2	34	F	23.1	30	oral cyclosporine, short episodes of oral glicocorticosteroids, topical steroids, and emollients
Patient 3	32	F	33.1	23	oral cyclosporine, UVB phototherapy, topical steroids, topical calcineurin inhibitors, and emollients
Patient 4	24	M	21.3	10	oral cyclosporine, short episodes of oral glicocorticosteroids, topical steroids, and emollients
Patient 5	85	M	20.6	10	oral cyclosporine, topical steroids, and emollients

**Table 2:** Treatment description and scheme of treatment monitoring.

Treatment	Dupilumab Dosing	Additional Therapy	Treatment Monitoring
Patient 1	- 200 mg of dupilumab subcutaneously every 2 weeks (after a loading dose of 400 mg)	- topical calcineurin inhibitors in case of flare-ups - emollients	- before treatment implementation - after first, second, and third doses of s.c. dupilumab (weeks 0, 2, 4, and 6)
Patient 2	- 300 mg of dupilumab subcutaneously every 2 weeks (after a loading dose of 600 mg)	- topical calcineurin inhibitors in case of flare-ups - emollients	- before treatment implementation - after first, second, and third doses of s.c. dupilumab (weeks 0, 2, 4, and 6)
Patient 3	- 300 mg of dupilumab subcutaneously every 2 weeks (after a loading dose of 600 mg)	- topical calcineurin inhibitors in case of flare-ups - emollients	- before treatment implementation - after first, second, and third doses of s.c. dupilumab (weeks 0, 2, 4, and 6)
Patient 4	- 300 mg of dupilumab subcutaneously every 2 weeks (after a loading dose of 600 mg)	- topical calcineurin inhibitors in case of flare-ups - emollients	- before treatment implementation - after first, second, and third doses of s.c. dupilumab (weeks 0, 2, 4, and 6)
Patient 5	- 300 mg of dupilumab subcutaneously every 2 weeks (after a loading dose of 600 mg)	- topical calcineurin inhibitors in case of flare-ups - emollients	- before treatment implementation - after first, second, and third doses of s.c. dupilumab (weeks 0, 2, 4, and 6)

## Data Availability

The original contributions presented in the study are included in the article, further inquiries can be directed to the corresponding author.
